# Improving the Efficiency for the Production of Bis-(2-Hydroxyethyl) Terephtalate (BHET) from the Glycolysis Reaction of Poly(Ethylene Terephtalate) (PET) in a Pressure Reactor

**DOI:** 10.3390/polym13091461

**Published:** 2021-04-30

**Authors:** Eider Mendiburu-Valor, Gurutz Mondragon, Nekane González, Galder Kortaberria, Arantxa Eceiza, Cristina Peña-Rodriguez

**Affiliations:** 1 ‘Materials+Tecnologies’ Research Group, Chemistry and Environmental Engineering Department, Faculty of Engineering, Gipuzkoa, University of the Basque Country (UPV/EHU), Plaza Europa 1, 20018 Donostia, Spain; eider.mendiburu@ehu.eus (E.M.-V.); gurutz.mondragon@ehu.eus (G.M.); galder.cortabarria@ehu.eus (G.K.); arantxa.eceiza@ehu.eus (A.E.); 2 Antex. Josep Hereu i Aulet 8, 17160 Anglés, Spain; nekane@sequal.com

**Keywords:** PET, glycolysis, BHET, pressure reactor

## Abstract

The depolymerization process of PET by glycolysis into BHET monomer is optimized in terms of reaction temperature and time, by carrying out the process under pressure to be faster for reducing the energy required. Almost pure BHET has been obtained by working in a pressure reactor at 3 bar both at 220 and 180 °C after short reaction times, while for longer ones a mixture of oligomers and dimers is obtained. Depending on the potential application required, the obtention of different reaction products is controlled by adjusting reaction temperature and time. The use of a pressure reactor allows work at lower temperatures and shorter reaction times, obtaining almost pure BHET. To the best of our knowledge, except for microwave-assisted procedures, it is the first time in which pure BHET is obtained after such short reaction times, at lower temperatures than those usually employed.

## 1. Introduction

Poly(ethylene terephthalate) (PET) is a semi-crystalline aromatic-aliphatic thermoplastic polyester used in food packaging manufacture, mainly for bottles (51%) and other food packaging (9.1%), as sheets and films (13.8%), as well as for non-food products (6.1%) [1]. Due to the great flexibility and low density, as well as low cost, the consumption of this polyester has increased in recent years [1], reaching to a demand of around 4 million tons in Europe during 2018 [2]. Among the most widely used polymers for food and beverage packaging, PET is the most suitable thermoplastic because its high mechanical, physical and chemical properties, including low gas transmission permeability [3,4]. The most important sources of PET residues are the manufacturing waste and post-consumer urban residues, which should be managed and recycled by specific companies.

The three main processes for the valorization of PET residues are the mechanical, chemical, and energetic recycling ones. Several companies around the world recycle PET by mechanical processing of post-consumer residues, mainly bottles, to produce laminates for packaging or fibers [5]. Among fibers, those with diameters from 5 to 150 mm (staple grades) are the most interesting, while larger ones are used to stuff anoraks, sleeping bags, and soft toys. Recycled PET is also used to spin fibers with lower diameter, which are then woven into “polar” fleece fabric used for sweatshirts, jackets and scarves [5]. PET urban waste show rheological, physical, and chemical properties very close to those of the virgin material, allowing their successful recycling by mechanical processes.

The environment provokes structural changes in PET samples due to degradation and changes in crystallinity or chain conformation [6,7,8]. These changes could be related to hydrolytic degradation, since hydrolysis is the main process occurring at temperatures below the glass transition temperature (T_g_) [9]. In addition, the hydrolysis of PET is an autocatalytic reaction that can occur during extrusion process [10] with the formation of carboxylic acids. To increase the life cycle of this material, several researchers and companies are working for the optimization of chemical recycling processes that allow the use of the most degraded PET waste such as marine litter or manufacturing residues [11].

The different methods for the chemical recycling of PET could be categorized into five main branches according to the reaction mechanism, including glycolysis, methanolysis, hydrolysis, ammonolysis and aminolysis [12]. The most attractive method is the chemical recycling by glycolysis, because it is considered to be a versatile chemical recycling method in which, besides the bis(2-hydroxyethyl) terephthalate (BHET) monomer, specialized oligomeric products such as polyols are also produced [13]. These sub-products can be further used for the synthesis of polymers such as unsaturated polyesters, polyurethanes, vinyl esters, or epoxy resins, among others [14,15].

To optimize the reaction rate and BHET monomer yield, four glycolysis methods have been developed. The solvent-assisted glycolysis, in which organic solvents as xylene constitute the reaction medium [16]; the supercritical glycolysis, performed at 450 °C and 7–15 MPa of pressure [17]; the microwave-assisted glycolysis, in which the maximum BHET yield could be obtained in a significantly lower reaction time (30 min) when compared to the 8–9 h reaction by conventional heating process [18]. Finally, in the catalyzed glycolysis, with ionic liquids as reaction medium, the reaction takes 5–10 h at 165–190 °C [19].

Among the benefits of above presented glycolysis methods, several works have defined that catalyzed with zinc acetate as the most effective [20,21,22,23], obtaining yields of 70% with a PET/EG molar ratio of 1:7.6, in 1 h at 196 °C in a three necks glass reactor under atmospheric pressure [22]. Conversions of almost 100% can be obtained with reactions times from 1 to 4 h [24]. The effect of temperature, PET/EG ratio and pressure on glycolysis have been analyzed by Chen et al. [25,26]. They confirmed that the depolymerization of PET materials depends on temperature and PET/EG ratio, occurring faster than at atmospheric pressure. Campanelli et al. [27] examined the reaction in a pressure reactor at temperatures above 245 °C, with a molar ratio PET/EG of 3:1, also concluding that depolymerization was faster under pressure. Therefore, it can be concluded that the pressure improves the depolymerization reaction of PET obtaining a higher amount of BHET. Therefore, several parameters can be modulated to obtain high yields, but also analyzing the composition of reaction products to define the most useful application or the most suitable polymer into which they will be transformed, by using proper conditions and reactants. Table 1 presents a comparative of the different glycolysis procedures found in the literature with that employed in the present work.

The aim of this work is the characterization of reaction products obtained from the chemical glycolysis of PET catalyzed by zinc acetate at different reaction times, to optimize the conditions for obtaining higher yields. Moreover, to develop an eco-friendlier process, a closed pressure reactor has been used to avoid the loss of organic volatile compounds, also reducing reaction times and temperatures in order to reduce the energy consumption of the recycling process.

## 2. Materials and Methods

Virgin PET pellets with a melt flow index of 61 ± 14 g/10 min and an intrinsic viscosity (I.V.) of 0.58 dL/g was kindly supplied by PlastiVerd (Barcelona, Spain). Ethylene glycol (EG) from Sigma-Aldrich (EEUU) was used as solvent for the glycolysis, with chemically pure zinc acetate PROBUS, C. Busquets (Badalona, Spain) as catalyst. For characterization purposes, standard BHET from Sigma-Aldrich (San Louis, MO, USA) and tetrahydrofuran (THF) provided by Macron Fine Chemicals™ (Avantor, Gliwice, Poland) were also used. Reagents were used as purchased without further purification.

### 2.1. Glycolysis Reaction

Glycolysis reactions were carried out under a pressure of 3 bar in a 0.3 L Parr 50 mini reactor equipped with a thermometer, a manometer, and a refrigeration system. Mechanical stirring was maintained constant at 1000–1200 rpm. A PET/EG weight ratio of 1:3 was employed (40 g PET, 120 g EG), with 1% mass of zinc acetate as catalyst, the working volume is approximately 160 mL. To analyze reaction kinetics at 220 °C, 8 different reaction times between 10 and 180 min (10, 20, 30, 40, 50, 60, 90, and 180 min) were chosen. Moreover, to evaluate the effect of temperature, depolymerizations were carried out at 180 and 200 °C and different reaction times of 30, 60, 90, and 180 min. All reactants were first placed into the reactor and then it was heated to the specific temperature and once the temperature was reached, the reaction time was quantified. The previous warm-up time was variable between 30–45 min.

**Scheme 1:**
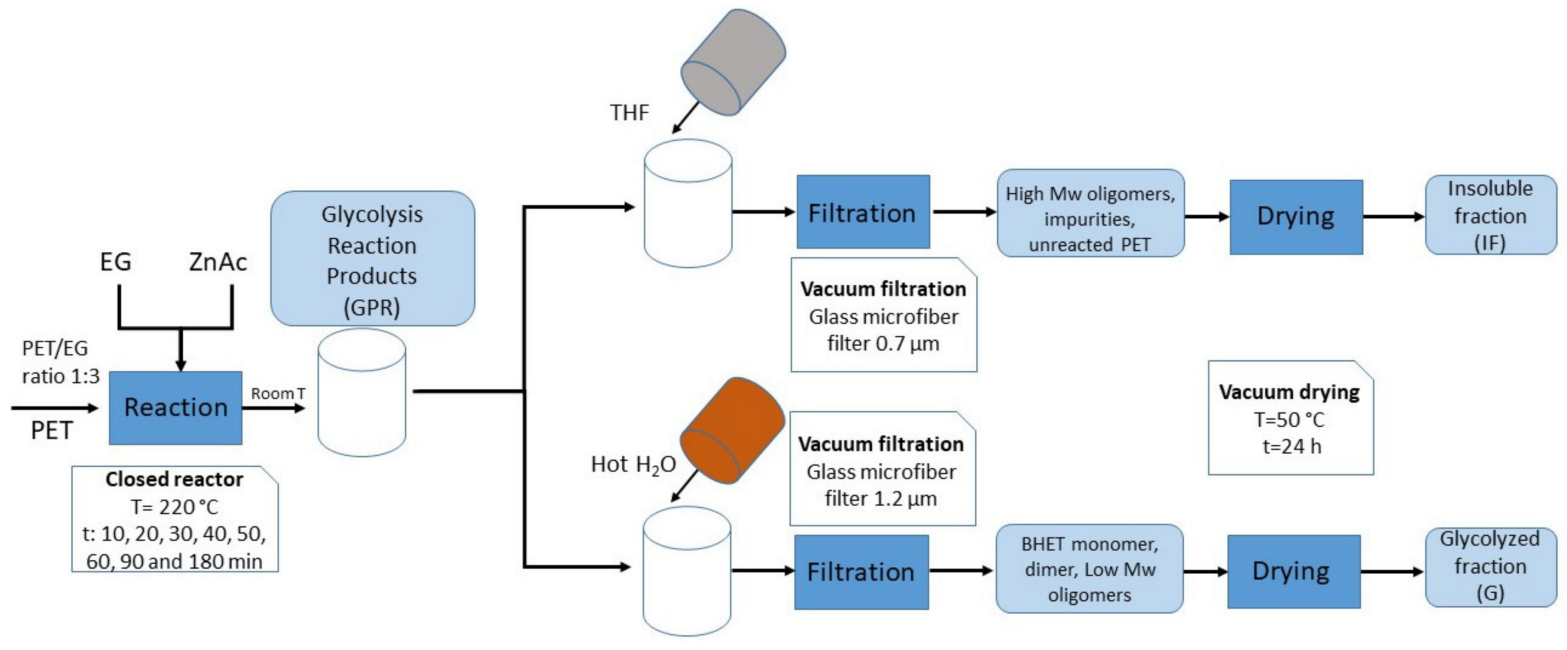
PET glycolysis reaction and products purification before characterization.

The process is summarized in Scheme 1. At the end of each reaction, the obtained liquid mixture was identified as the glycolysis reaction product (GRP). To determinate the unreacted PET and high molecular oligomers, THF filtration was performed, separating glycolysis products soluble in THF, such as BHET monomers, dimers or low molecular weight oligomers, from insoluble fractions (IF). In the other hand, glycolyzed products (G) were determined by filtration. With this purpose, a hot water excess was used to solve the GRP and to ensure a better separation between glycols, BHET and oligomers [22]. Then, samples were cooled at room temperature during 24 h, for being then stored in a refrigerator for 15 min at 4 °C to induce the precipitation of white crystals [28] of BHET monomer and other oligomeric fractions. Both the insoluble (IF) and the glycolyzed fractions (G) were filtrated by vacuum filtration using glass microfiber filters with pore diameters of 0.7 and 1.2 µm, respectively. Finally, the filters were dried under vacuum at 50 °C for 24 h.

The yield (η) for BHET was calculated by weight difference as shown in the following equation.

(1) $$\eta \left(\mathrm{weight}\%\right)=\frac{W_{\mathrm{BHET}.f}/{\mathrm{MW}}_{\mathrm{BHET}}}{W_{\mathrm{PET}.0}/{\mathrm{MW}}_{\mathrm{PET}}}\times 100$$

where W_PET.0_ refers to the initial PET weight and W_BHET.f_ to the BHET weight obtained at the end of the process. Moreover, Mw_BHET_ and Mw_PET_ are the molecular weights of BHET (254 g/mol) and PET (192 g/mol) repeating unit, respectively [18,22].

### 2.2. Characterization Techniques

Weight and number average molecular weights, *M*_w_ and *M*_n_, were determined by gel permeation chromatography (GPC, Thermo Fisher Scientific, Waltham, MA, USA) using a Thermo Scientific chromatograph, equipped with an isocratic Dionex UltiMate 3000 pump and a RefractoMax 521 refractive index detector. The separation was carried out at 30 °C within four Phenogel GPC columns from Phenomenex with 5 µm particle size and porosities of 10^5^, 10^3^, 100, and 50 Å, located in an UltiMate 3000 thermostated column compartment. Tetrahydrofuran (THF) was used as mobile phase at a flow rate of 1 mL/min. Samples were prepared solving obtained BHET into THF at 1 wt.% and filtering using nylon filters with 2 µm pore size. Weight average and number average molecular mass, *M*_w_ and *M*_n_ respectively, were reported as weight average based on calibration curve with monodisperse polystyrene standards.

The characteristic functional groups and chemical interactions from PET, BHET and glycolyzed products were analyzed by Fourier transform infrared spectroscopy (FTIR, Thermo Fisher Scientific, Waltham, MA, USA). Spectra were recorded by a Nicolet Nexus spectrometer provided with a MKII Golden Gate accessory (Specac) with a diamond crystal at a nominal incidence angle of 45° and ZnSe lens. Spectra were recorded in attenuated total reflection (ATR) mode between 4000 and 500 cm^−1^, performing 64 scans with a resolution of 8 cm^−1^.

Thermogravimetric analysis (TGA, Columbus, OH, USA) was performed on a TGA/SDTA851 Mettler Toledo equipment to evaluate the thermal stability of glycolyzed fractions. Samples were heated from room temperature to 800 °C at a heating rate of 10 °C min^−1^ under a nitrogen atmosphere.

Thermal properties of PET materials were determined by differential scanning calorimetry (DSC, Columbus, OH, USA), performed using a Mettler Toledo DSC3+ equipment provided with a robotic arm and an electric intracooler as refrigerator unit. 5–10 mg of sample were encapsulated in aluminum pans and heated from 25 to 170 °C at a heating rate of 10 °C min^−1^ in nitrogen atmosphere, determining the melting temperatures (T_m_) of G products.

## 3. Results and Discussion

**Scheme 2:**
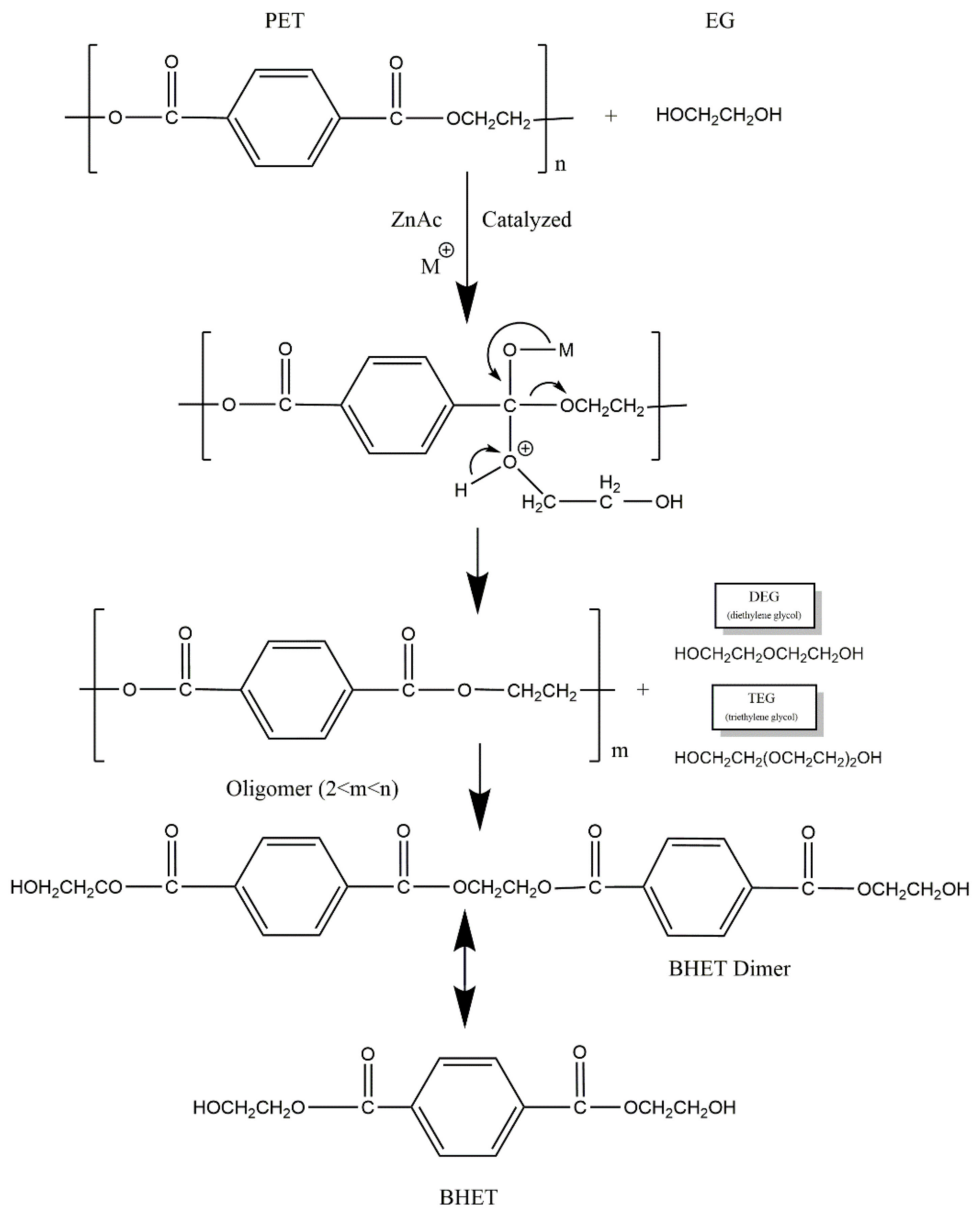
Mechanism for glycolysis reaction [29,30].

The depolymerization of PET by glycolysis is the reverse of the polycondensation reaction for PET synthesis (Scheme 2). As a result, the glycolysis product is a heterogenous mixture of BHET, dimmers, and other oligomers. Reaction yield (ƞ) and insoluble fraction (IF) values obtained at different reaction times are summarized in Table 2.

Reaction yield reaches the 55% at only 10 min of reaction, increasing up to almost 70% after 30 min. For longer reaction times, similar or lower yields are obtained (except that for 90 min, very close to 70%). Therefore, it can be assumed that the optimal reaction time for obtaining the highest yield of reaction will be between 30–90 min. This study was completed by GPC, as will be shown below.

**Figure 1:**
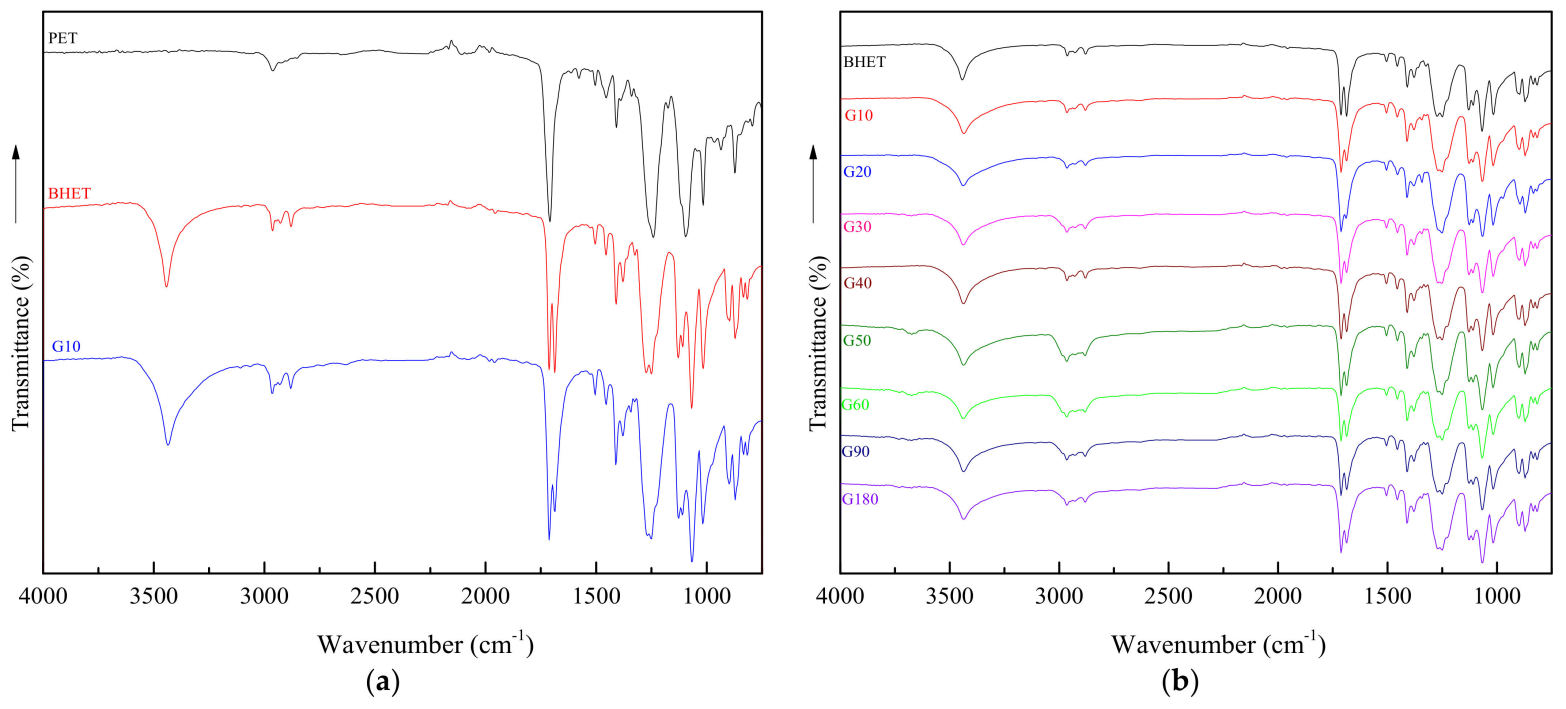

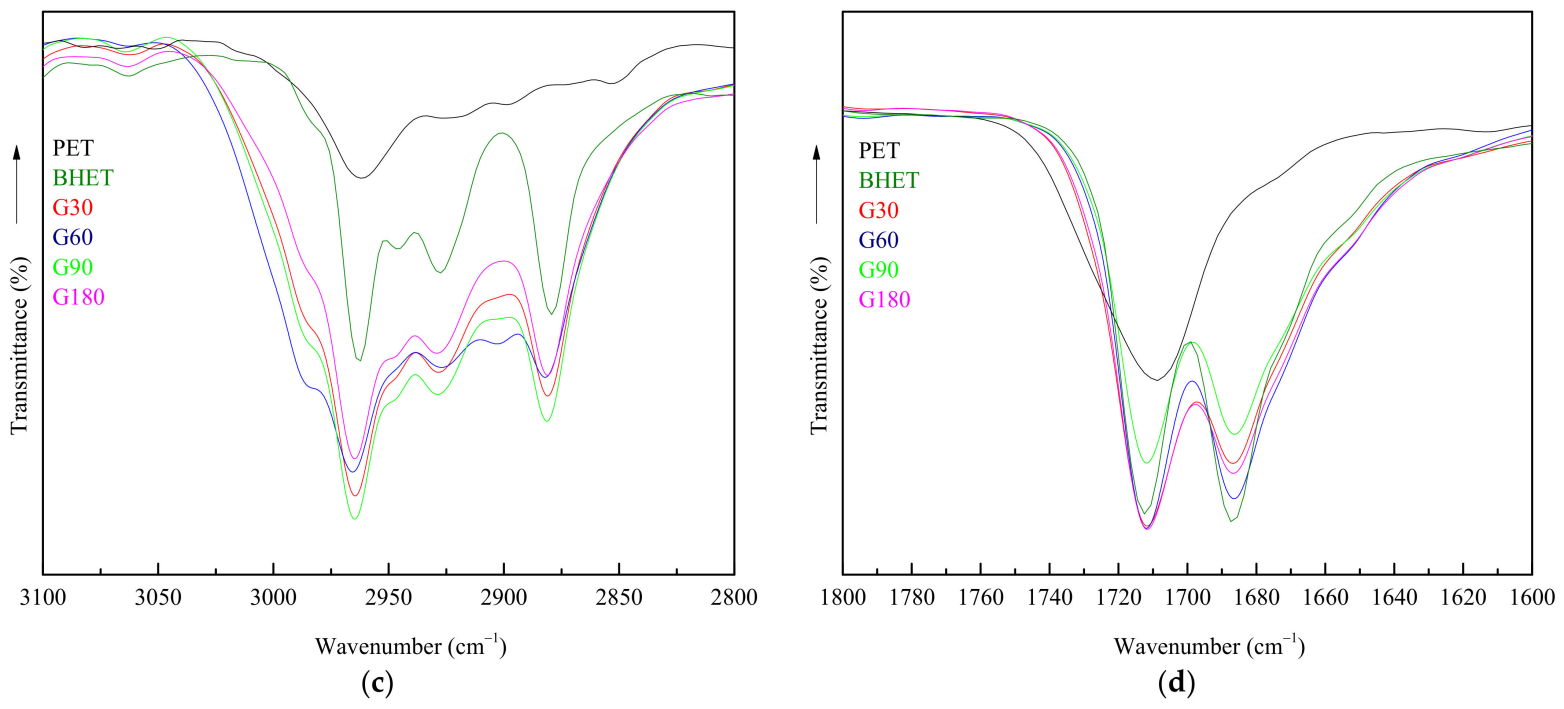
FTIR spectra of PET and reaction products: (**a**) FTIR spectra of reference PET and BHET together with that of the product obtained after 10 min of reaction; (**b**) FTIR spectra of products obtained at different reaction times together with that of the reference BHET; (**c**) Band corresponding to aliphatic groups –CH_2_–, for G products, BHET and PET; (**d**) Band corresponding to ester groups C=O, for G products, BHET and PET.

To determinate the composition of the final product, FTIR, DSC, and GPC analysis were performed. Figure 1 shows FTIR spectra of glycolyzed products (G fraction) obtained from reaction, as well as those of reference PET and BHET.

As can be seen in Figure 1a, the spectrum of the product obtained after 10 min of reaction matches with that of BHET. PET depolymerization leads to the formation of hydroxyl groups, reflected in the band at 3600–3200 cm^−1^, present at the spectra of BHET and G10 [29]. The strong absorption band corresponding to the ester group at 1712 cm^−1^ is also detected. From Figure 1b, showing the spectra of samples obtained at different glycolysis times, can be seen that there are no significant differences among different materials.

FTIR spectra with higher magnification, showing the most representative bands related to characteristic functional groups are also presented in Figure 1 for a deeper analysis. From Figure 1c is observed that at 3100–2800 cm^−1^ interval, two peaks corresponding to the aliphatic (–CH_2_–) appear for G samples while only a single peak is detected in the PET spectra. The presence of the double peak is related to the BHET monomer while the single peak is related to the presence of BHET oligomers [31], only present at PET samples. In the same way, the band related to the ester group at 1712 cm^−1^ appears at PET spectrum as a single peak, while a double one is detected for the rest of samples, probably related with the amorphous and crystalline parts present in each sample [32].

**Figure 2:**
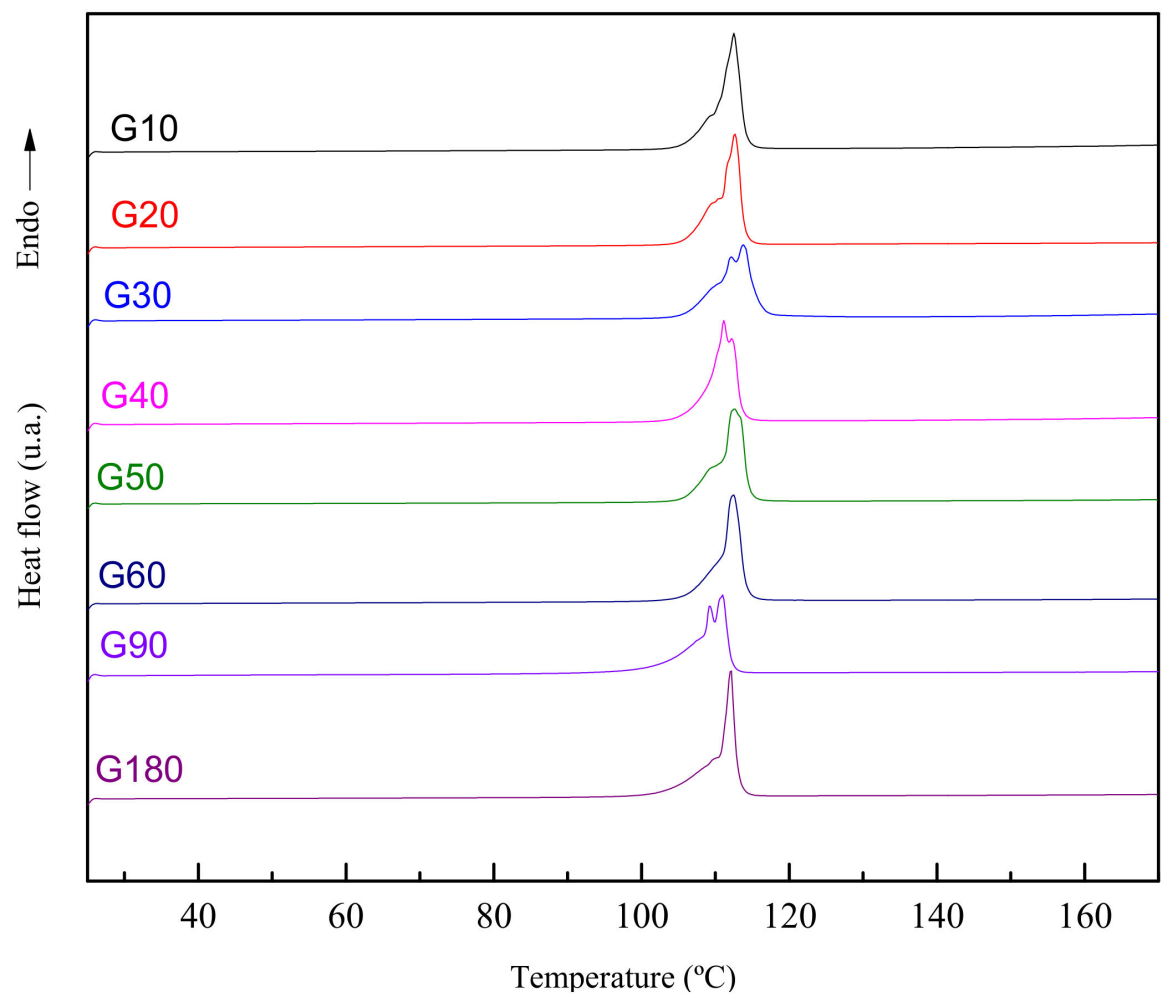
DSC thermograms corresponding to the products obtained at different reaction times.

DSC thermograms of the products obtained at different reaction times can be seen in Figure 2, corresponding to a heating scan from 25 to 170 °C at a heating rate of 10 °C/min.

All thermograms show the maximum peak corresponding to the melting temperature at around 100–110 °C, matching with that found for BHET in the literature [31], thus meaning that for all reaction times the product obtained is mainly BHET.

**Figure 3:**
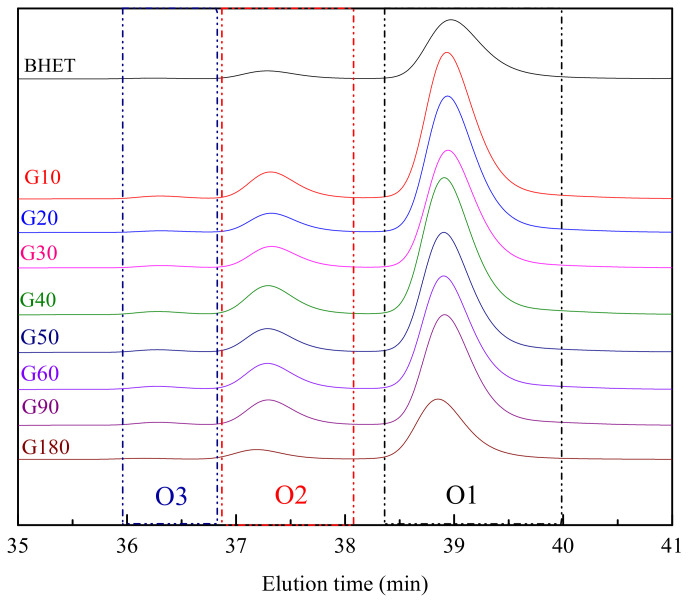
GPC traces for reaction products obtained at different times, together with that of BHET.

Molar mass values for obtained products have been measured by GPC. Figure 3 shows GPC traces obtained for the reaction products obtained at different reaction times, together with that corresponding to BHET. Obtained oligomer contents, integrating the area under the peak, and their Mw and Mn are summarized in Table 3, where O1, O2, and O3 correspond to the fraction of BHET monomer, dimer, and oligomer, respectively, with retention times of 39, 36 and 37 min [33].

Among the three different signals shown, that corresponding to O1 is the most significant one, with a molar mass of 162–171 g/mol (*M*_w_), related to BHET monomer [31]. Peaks with lower intensity appear around 36–38 min, related to BHET oligomers with 2–3 repeating units: O2 fraction with a corresponding molar mass of 362–384 g/mol (*M*_w_), and O3 one with a molar mass of 559–600 g/mol (*M*_w_).

**Figure 4:**
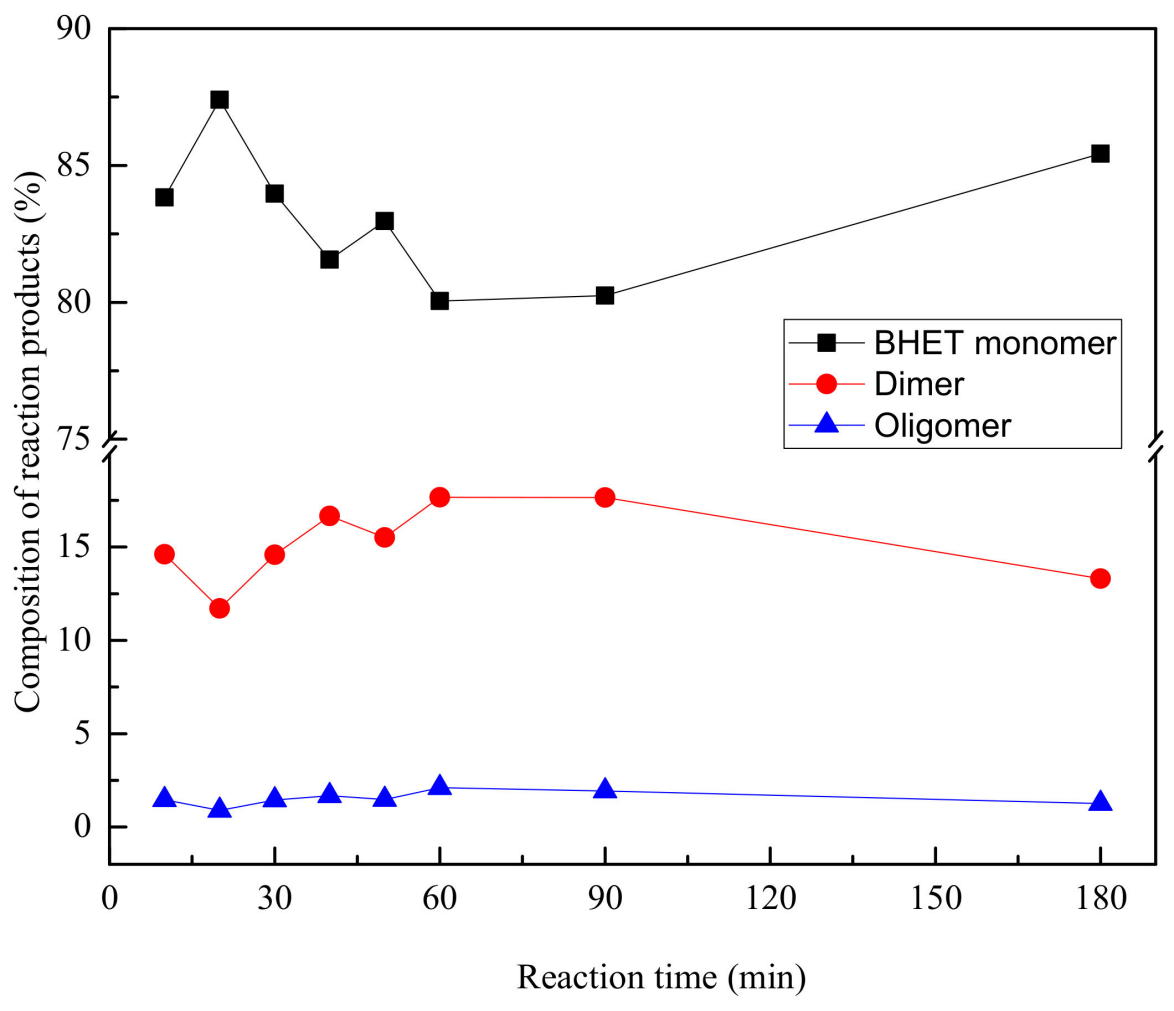
Fractions of different compounds as obtained by GPC at different reaction times.

As can be concluded from Table 3, the highest amount of BHET monomer is obtained after 20 min of reaction. The presence of dimer seems to be quite constant, while oligomer fraction is almost inexistent, as can be also seen in Figure 4, showing the fractions obtained with reaction time.

**Scheme 3:**
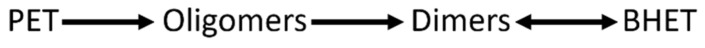
Reversible PET glycolysis reaction.

Monomer and dimer BHET fractions do not show a regular trend, increasing and decreasing for different reaction times, probably related to the fact that PET glycolysis is a reversible reaction, as shown in Scheme 3 [33].

It seems that PET depolymerization into dimer occurs in a relatively short time interval, depolymerization rate of dimer into BHET constituting an important parameter [33].

**Figure 5:**
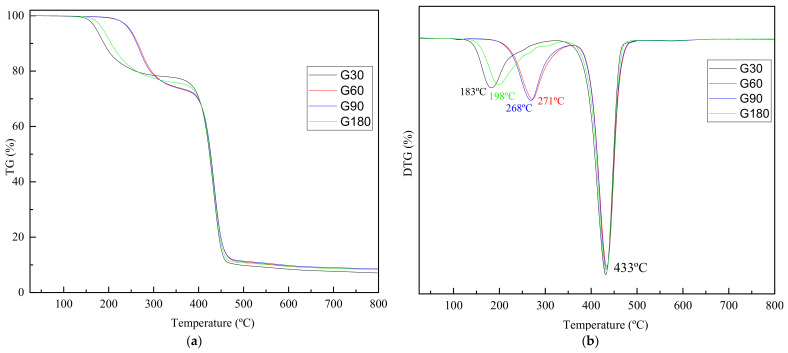
TGA (**a**) and DTGA (**b**) curves for samples obtained at different reaction times.

Regarding TGA analysis of different glycolyzed fractions Figure 5 shows both TGA and DTGA curves for all samples obtained, while main parameters are summarized in Table 4.

Two main mass losses are observed for all samples (Figure 5a). The highest mass loss occurs around 433 °C, constituting approximately the 65% of mass loss, related to the thermal degradation of PET that has been produced in the same thermogravimetric analysis process by the thermal polymerization of BHET [34]. The first loss of mass, a loss of 21–27%, is related to the thermal degradation of the monomer and oligomer fraction [34]. From the derivative curves of Figure 5b it is observed that for G30 and G180, the mass loss step occurs at lower temperatures than for G60 and G90. It could be attributed to the evolution of dimers or oligomers with low molar mass, as was previously seen by GPC, G60, and G90 presenting higher dimer fractions. Therefore, it can be deduced that the thermal degradation above 200 °C is related to dimers or oligomers with low molar mass, while if it occurs at lower temperatures is related with BHET monomer [29,31,35].

**Figure 6:**
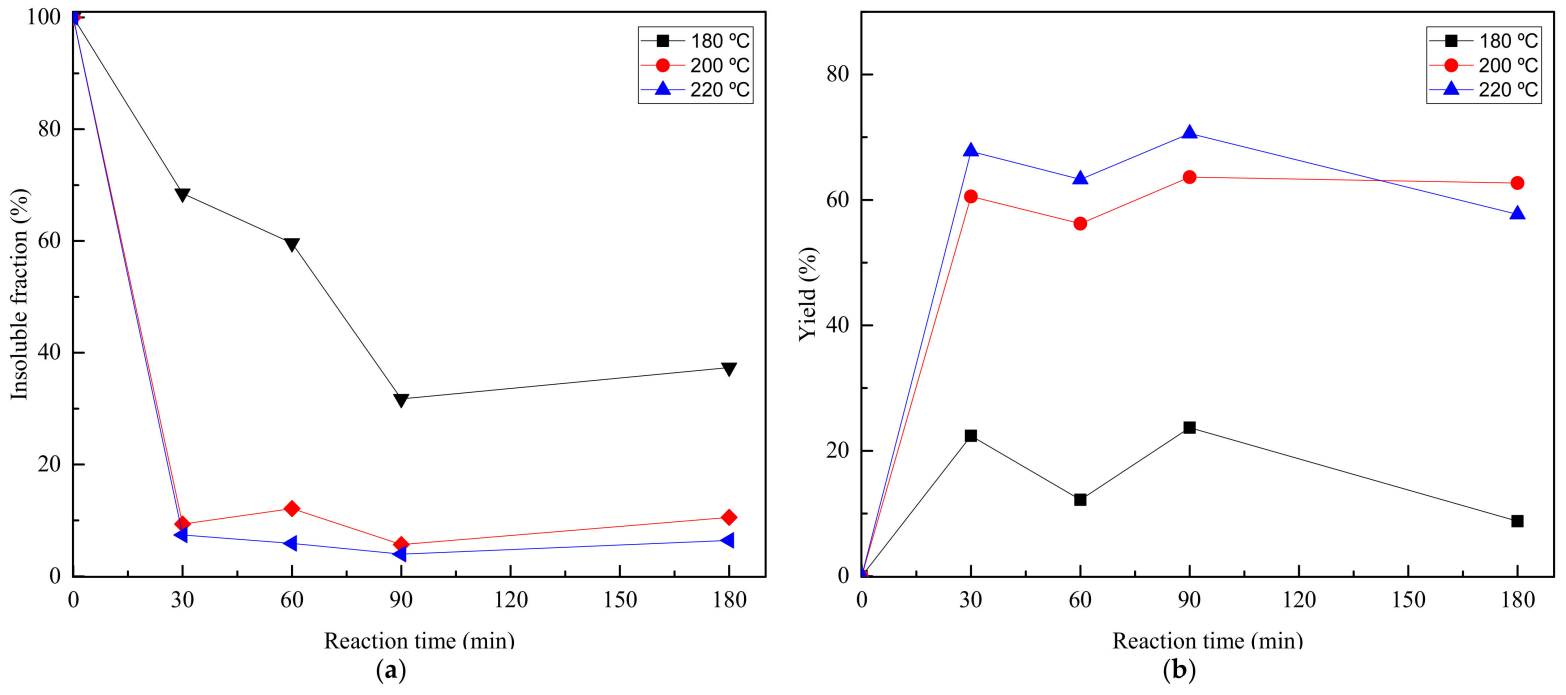
Reaction yield (**a**) and insoluble fraction (**b**) values vs. reaction time at 180, 200 and 220 °C.

Finally, the effect of temperature on the glycolysis process has been analyzed, by performing glycolysis reactions at 180, 200 and 220 °C, for 30, 60, 90, and 180 min. Obtained reaction yields and insoluble fractions are shown in Figure 6.

**Figure 7:**
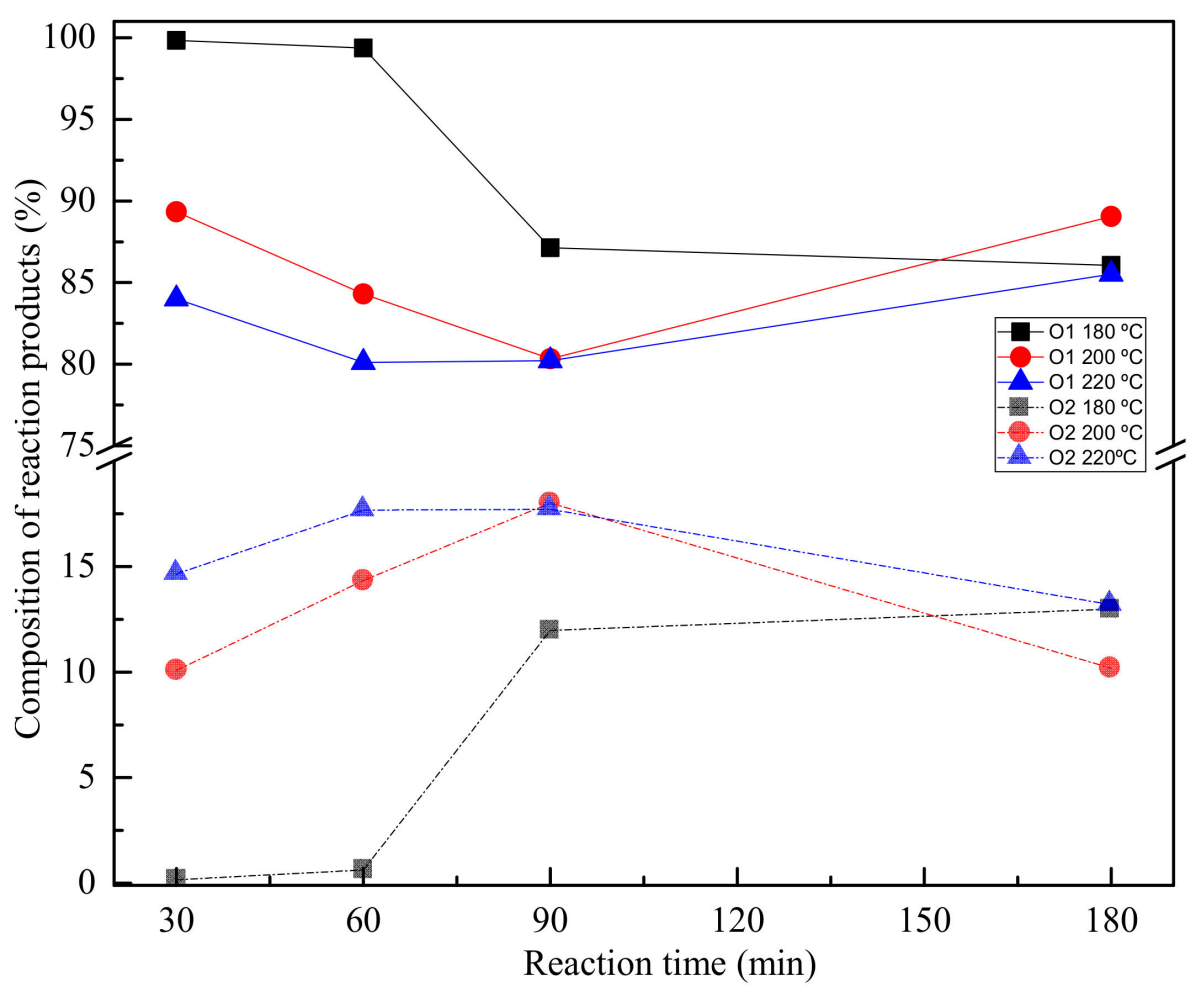
BHET monomer and dimer fraction values of reaction compounds at different reaction temperatures and times, as calculated by GPC.

Regarding insoluble fraction (Figure 6a), for 200 and 220 °C, after 30 min of reaction, is lower than 10%, while for the reaction at 180 °C the insoluble faction is considerably higher. As it can be seen in Figure 6b, above 30 min of reaction the BHET yield decreases for all the samples. This could be because the depolymerization reaction is reversible, therefore at 60 min of reaction the polymerization can occur, generating dimers or oligomers, as can be seen from GPC analysis shown in Figure 7. Temperature is a critical factor to take into account, as seen by results presented at 180 °C, at which for short reaction times as 30 and 60 min, the percentage of unreacted PET is 67% and 55%, respectively, considerably higher than those at higher temperatures.

Figure 7 shows BHET monomer and dimer fractions from products obtained at different temperatures and reaction times, as obtained by GPC.

**Figure 8:**
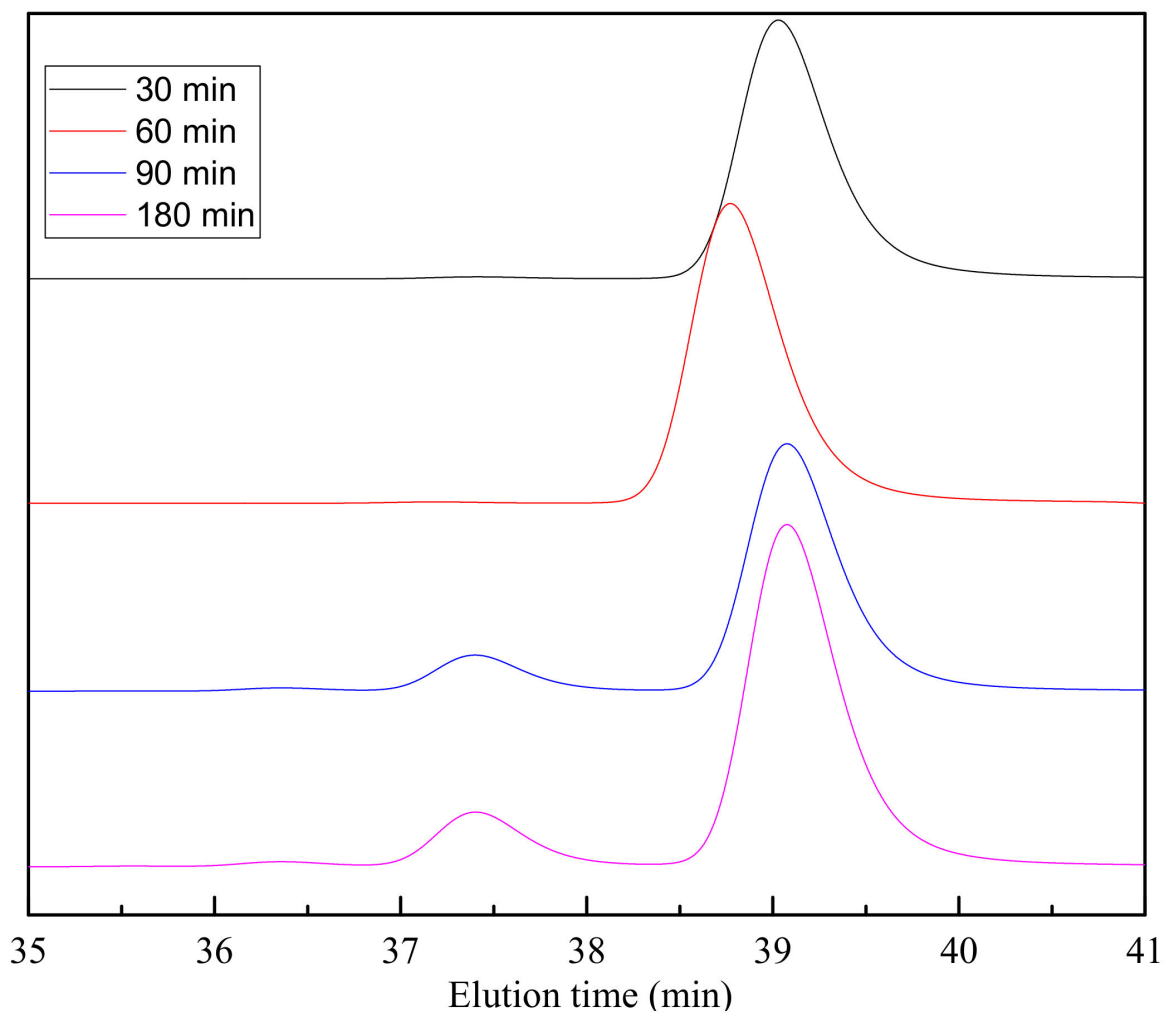
GPC results for the depolymerization at 180 °C for different reaction times.

Observing the values obtained for the reactions at different temperatures after 60 min, an upward trend is observed for the amount of dimer in the sample, confirming that after 60 min of reaction, the BHET polymerized being the oligomer fraction higher and the BHET fraction lower. Therefore, it can be stated that during glycolysis reaction, polymerization reaction also occurs, since it is an easily reversible reaction. From GPC analysis, it can be also concluded that for lower reaction times, samples with lower dispersity of molar mass are obtained, indicating the higher purity of BHET monomer. In that conditions, a single peak is observed in the GPC from Figure 8 at 39 min that is related with the monomer of BHET.

## 4. Conclusions

The depolymerization process of PET depends on temperature and reaction time and those parameters must be adjusted for optimizing the process. It has been shown that after 10 min of reaction at 220 °C under pressure, the final product obtained is mostly BHET. However, as reaction time increases, dimers and low molecular weight oligomers are also generated by the polymerization process occurring due to the reversibility of the depolymerization reaction of PET into BHET.

On the other hand, pure BHET has also been obtained in this work by working under pressure at 180 °C and 30 and 60 min of reaction, implying that depolymerization can also occur at lower temperatures and reaction times. For longer reaction times, the yield of BHET increases but the purity is lower, as the number of dimers and oligomers also increases. It has been seen that there is a direct relationship between the yield of BHET, the quantity of dimers and monomers analyzed by GPC, and the degradation temperatures: a higher fraction of dimer in the final product results into a higher degradation temperature.

The conditions of the depolymerization reaction of PET residue becomes of crucial importance, since depending on reaction conditions, products with different compositions and potential applications can be obtained. The use of a pressure reactor allows work at lower temperatures and reaction times, reducing the consumed energy during the process and in consequence, the environmental impacts of chemical recycling.

## Figures and Tables

**Table 1:** Comparative between depolymerization procedures found in the literature and other present work.

Reference	Method	Time (h)	Temperature (°C)	Pressure (at)	Catalyst	Ratio PET:EG	Yield (%)
[16]	Glycolysis in xylene	1–3	170–245	-	-	1:0.5–1:3 Molar ratio	20
[18]	Glycolysis by microwave irradiation	0.5	180–190	-	ZnAc 0.5 %	1:2,1:4, 1:6 Molar ratio	40
[19]	Ionic liquids as reaction medium	5–10	160–195	1	Ionic liquids 1–4 L	1:4 (*w*/*w*)	Max Yield 78 Conversion 70
[20]	Glycolysis in a pressure reactor		235–275	High pressure	ZnAc	1:1.3 (*w*/*w*)	-
[21]	Autoclave reactor	1	260	5	ZnMn_2_O_4_ 1 %	5:86 Molar ratio	92.2
[22]	Glycolysis at atmospheric pressure	1–2	196	1	ZnAc 1 % Or Na_2_CO_3_ 1%	1:7.6 Molar ratio	70
[23]	Glycolysis in potassium nitrate bath	8	190	-	Metal acetate	1:4 Molar ratio	-
[24]	Glycolysis at atmospheric pressure	1–4	180–190	1	ZnAc 2.35 %	1:4 (*w*/*w*)	91.6
[25]	Glycolysis under pressure	0.5–3	190–240	1–6.12	-	1:1–1:4 Molar ratio	-
[26]	Glycolysis at atmospheric pressure	2–10	190	1	ZnAc 0.25–0.75 %	1:4 Molar ratio	-
Our work	Glycolysis under pressure	10–180	220	3 bar	ZnAc 1 %	1:3 (*w*/*w*)	55–71

**Table 2:** Insoluble fraction and yield values obtained at 220 °C at several reaction times.

Fraction	Insoluble (%)	ƞ (%)
G10	5.78	55.00 ± 10
G20	8.03	59.67 ± 14
G30	7.39	67.75 ± 9
G40	5.13	58.34 ± 4
650	5.47	62.95 ± 6
G60	5.88	63.29 ± 3
690	3.97	70.60 ± 9
G180	6.41	57.71 ± 6

**Table 3:** Oligomer content (%) and *M*_w_ and *M*_n_ values as obtained by GPC.

Samples	O1 *M*_n_ = 155–167 g/mol *M*_w_ = 160–171 g/mol (%)	O2 *M*_n_ = 358–379 g/mol *M*_w_ = 362–384 g/mol (%)	O3 *M*_n_ = 556–595 g/mol *M*_w_ = 559–600 g/mol (%)
G10	83.83	14.61	1.46
G20	87.40	11.71	0.89
G30	83.97	14.58	1.45
G40	81.56	16.67	1.67
G50	82.97	15.51	1.47
G60	80.05	17.66	2.11
G90	80.25	17.65	1.93
G180	85.43	13.31	1.26

**Table 4:** Main parameters obtained by thermogravimetric analysis.

Samples	1. Step		2. Step	
Time (Min)	T_1_ (°C)	Mass Loss (%)	T_2_ (°C)	Mass Loss (%)
30	183	22.62	432	70.27
60	271	27.31	434	64.4
90	267	26.77	433	64.68
180	198	24.41	433	67.29

## Data Availability

The data presented in this study are available on request from the corresponding author.
